# Diagnostic accuracy of cerebrospinal fluid liquid biopsy and MRI for leptomeningeal metastases in solid cancers: A systematic review and meta-analysis

**DOI:** 10.1093/noajnl/vdad002

**Published:** 2023-03-05

**Authors:** Yoko Nakasu, Shoichi Deguchi, Satoshi Nakasu, Mutsumi Yamazaki, Akifumi Notsu, Koichi Mitsuya, Nakamasa Hayashi

**Affiliations:** Division of Neurosurgery, Shizuoka Cancer Center, Shizuoka, Japan; Department of Neurosurgery, Shiga University of Medical Science, Shiga, Japan; Division of Neurosurgery, Shizuoka Cancer Center, Shizuoka, Japan; Division of Neurosurgery, Omi Medical Center, Shiga, Japan; Medical Library, Shizuoka Cancer Center, Shizuoka, Japan; Clinical Research Center, Shizuoka Cancer Center, Shizuoka, Japan; Division of Neurosurgery, Shizuoka Cancer Center, Shizuoka, Japan; Division of Neurosurgery, Shizuoka Cancer Center, Shizuoka, Japan

**Keywords:** cell, free tumor DNA, circulating tumor cell, diagnostic performance, leptomeningeal metastasis, meta, analysis

## Abstract

**Background:**

Cerebrospinal fluid (CSF) cytology remains the gold standard approach for diagnosing of leptomeningeal metastases (LM), but has clinical problems due to its low sensitivity. This systemic review and meta-analysis evaluated the diagnostic accuracy of the novel CSF biomarkers of liquid biopsy and magnetic resonance imaging (MRI) for detecting LM in patients with solid cancers.

**Methods:**

A systematic search of electronic databases was conducted to identify all published diagnostic accuracy studies on CSF liquid biopsies and MRI since January 2000 with registration for PROSPERO (#CRD42022301988). Articles were selected based on pre-defined inclusion and exclusion criteria following the PRISMA 2020 statement.

**Results:**

The search yielded 3790 citations, and 10 studies with 668 patients were included in the final analysis. The pooled prevalence of LM was 50.9% (340/668). The respective sensitivity and specificity for index tests were as follows: circulating tumor cells (CTC), 87.0% (95% confidence interval [CI] 77.9–92.6%) and 93.8% (86.9–97.2%); cell-free tumor DNA, 97.9% (19.3–100%) and 89.0% (25.3–99.5%); MRI 59.4% (60.7–76.9%) and 97.6% (77.3–99.8%); cytology, 71.9% (54.7–82.9%) and 100%. The diagnostic odds ratio was 100.6 (29.38–344.09) for CTC and 93.3 (88.42–1034.05) for MRI.

**Conclusion:**

Novel CSF liquid biopsies and MRI may offer improved diagnostic accuracy for LM from solid cancers; however, further research is required to specify the threshold values and to construct standards for individual primary cancers.

**Key Points**

- A meta-analysis of the diagnostic performance for leptomeningeal metastases.
- Solidifying the superior sensitivity of CTC and moderate specificity of MRI.
- Implications of early diagnoses with a biomarker-driven and less-invasive approach.

**Importance of the Study**

Despite the increasing clinical research on leptomeningeal metastases (LM) in patients with solid cancer, their utility in clinical practice depends on rigorous and generalizable evidence in the early initial diagnosis of the disease. This study systematically reviewed and evaluated the diagnostic performance of cerebrospinal fluid (CSF) liquid biopsy and advanced magnetic resonance imaging (MRI) for patients suspected of having LM under the PRISMA 2020 statement and using a hierarchical meta-analysis. This is the first meta-analysis of the diagnostic accuracy of liquid biopsy and MRI for LM. Circulating tumor cells (CTC) were superior to cytology regarding sensitivity, while MRI outperformed CTC regarding specificity. It would be beneficial to have a biomarker-driven and less-invasive approach to LM, as this would facilitate clinical trials for LM in the era of targeted therapy. Revised standards and guidelines for the initial diagnosis need to be established.

Leptomeningeal metastasis (LM) is a potentially catastrophic condition with an impaired quality of life and high mortality in the clinical oncology. LM is present in 2–12% of cancer patients at the time of initial intracranial involvement, but can develop in up to 37% later in the clinical course.^1^ Various solid cancers may provoke LM.^2^ The incidence of LM in patients with solid cancer might be underestimated,^3^ and it has become an increasingly common diagnosis as better treatment improvements lengthen the survival of patients with cancer.^4^

Early detection and the start of vigorous treatment can prevent the onset of irreversible symptoms and prolong the survival with an acceptable quality of life.^5–7^ However, the diagnosis is difficult, particularly in the early stages when treatment is most beneficial.^8^ The diagnosis of LM is currently based on the three examination modalities: neurological evaluations, neuroimaging and cerebrospinal fluid (CSF) cytology.^9,10^ However, the performance of these modalities is reportedly suboptimal.^5^ CSF cytology remains the gold standard for the diagnosis of LM, but has been of low sensitivity.^2,8^ Magnetic resonance imaging (MRI) has been reported to have a similar sensitivity to CSF examinations but a lower specificity.^5,11^

Recently, several studies have focused on introducing new potentially useful biomarkers with molecular information and quantitative data of malignant cells in the CSF.^5,12^ Liquid biopsies sample tumor-derived material released in the blood, CSF and other biofluids. The advantage of utility of liquid biopsies includes an early diagnosis and the ability to assess tumor heterogeneity and molecular changes.^13^ There has been an increasing interest in the application of liquid biopsies to neuro-oncological diagnoses. In addition, MRI has rapidly advanced in the production of high-quality images for the detection and accurate localization of the diseases with a non-invasive advantage.

The present study involved a systematic review and meta-analysis of the available literature concerning the diagnostic accuracy of liquid biopsies, including CSF circulating tumor cells (CTC) and cell-free tumor DNA (ctDNA). We compared the performances of a liquid biopsy and recent high-quality MRI techniques with that of cytology for the diagnosis of LM in patients with solid cancers. This study is the first meta-analysis concerning the diagnostic accuracy of liquid biopsies and MRI for LM.

## Methods

### Search Strategy

A systematic search in PubMed, Scopus and the Cochrane Library was conducted to identify all relevant literature published between January 2000 and March 2022. The search was initially performed by a librarian (M.Y.) in January 2022, and updated on March 1, 2022.

The search strategy can be found in the Supplementary Material. The citations of the included articles were hand-searched to identify additional articles. The review and meta-analysis were conducted in accordance with the Preferred Reporting Items for Systematic Reviews and Meta-Analysis statement (PRISMA 2020 statement).^14^ The protocol for this study was registered on PROSPERO: International prospective register of systematic reviews (2022, CRD42022301988) [https://www.crd.york.ac.uk/PROSPERO].

### Selection Criteria

The search was limited to publications written in English. Eligible studies were observational studies that assessed the diagnostic accuracy of the investigated CSF biomarkers and/or MRI in patients with suspected LM from solid cancers. LM was ideally confirmed by integrated clinical findings involving cytology, MRI, clinical neurological findings, and/or autopsy. Studies were included if they involved more than 20 patients with suspected LM from solid cancers.

A study was included in our meta-analysis if true positive (TP), false positive (FP), true negative (TN), and false negative (FN) test results were able to be derived to construct two-by-two contingency tables directly from published data.

The exclusion criteria were as follows: (1) studies reporting diagnostic methods only; (2) studies reporting pediatric population, pituitary tumors, hematologic cancers, and/or primary brain tumors; (3) case reports, abstracts or conference proceedings; (4) studies including patients following craniotomy for brain metastases; and (5) case-controlled studies.^15^

### Study Selection and Data Extraction

Extracted citations were imported into the Mendelay system site (https://www.mendeley.com/) for study selection. Following removal of duplicates, titles and abstracts were screened, and full texts of relevant publications were reviewed. Two authors (YN, SD) applied the pre-defined eligibility criteria and selected studies independently based on title and abstract, being blinded to another’s decisions. A third researcher (SN) checked the extracted data. Disagreements between individual judgments were resolved through discussions.

Study characteristics were extracted by the two independent reviewers (YN, SD) from included studies, with disagreements resolved through discussion. Extracted data extracted included the following: (1) Author, year, publishing; (2) type of study; (3) patient clinical features; (4) type of tumors studied; (5) index tests, technique, performed numbers; (6) reference tests; and (7) TP, FP, TN, and FN results. Data to construct two-by-two contingency tables were retrieved to calculate diagnostic accuracy.

### Assessing the Methodological Quality

Using modified criteria based on the Quality Assessment of Diagnostic Accuracy Studies 2 (QUADAS-2) tool, two reviewers (YN, SD) independently and critically appraised the selected articles for the risk of bias (ROB) and applicability.^16^ Judgments were discussed after which consensus was reached.

### Data Synthesis and Statistical Analyses

To provide diagnostic test accuracy results for oncological profession treating LM, the sensitivity, specificity, diagnostic odds ratio (DOR), receiver operating characteristic (ROC) curves, and scatterplots in ROC were calculated using the Review Manager 5.3 (RevMan-5) software program [https://revman.cochrane.org/].^17,18^

In addition, the Diagnostic Test Accuracy Meta-Analysis v2.01 (MetaDTA) online software program [https://crsu.shinyapps.io/dta_ma/] was used for the meta-analysis with a random-effect model.^19,20^ MetaDTA fits the random effects bivariate binominal model of Chu and Cole.^21^ The bivariate model is mathematically equivalent to the hierarchical summary ROC (HSROC) model.^22^ The HSROC parameters are estimated using the bivariate model parameters and the equivalence equations of Harbord et al.^22^ The HSROC parameters were used to draw the summary ROC (SROC) plot with a summary estimate point of sensitivity for index tests. Area under curve (AUC) and Partial AUC were calculated using R-package mada (R Foundation for statistical Computing, Vienna, Austria). Subgroup analyses were avoided when the number of studies in a group was smaller than five. Data from individual studies and pooled results were expressed as the means with 95% confidence intervals (CI). A *P* value of <.05 was considered statistically significant.

## Results

### Search Results

**Figure 1.**
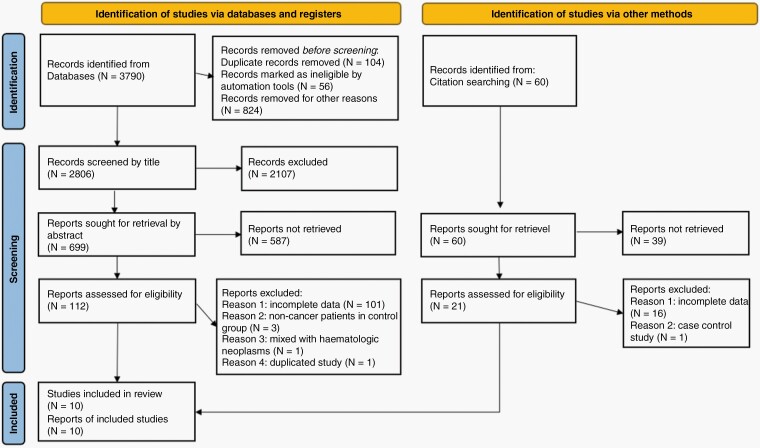
Flow diagram for the systematic review which included searches of databases, registers and other sources according to the PRISMA 2020 statement.

Figure 1 depicts the selection of articles included in this study according to the PRISMA 2020 statement.^14^ A total of 3790 citations were identified of which 112 were retrieved for full-text review. Duplicates were removed. Cross references of relevant review were screened. A total of 10 studies were ultimately selected for final critical appraisal.^5,6,23–30^Table 1 shows the characteristics of the included 10 studies evaluating 668 patients for LM. The primary cancers varied between studies, with breast cancer being the most common.

**Table 1.** Overview of the design and main findings of the 10 studies on the LM diagnosis included in the qualitative analysis

Study (year)	Study design, country, study period	Primary cancers, No.	Number of patients with LM/suspected LM	Patient Age (years), Men:Women	Index test, number of examined patients	Reference tests	Outcomes for the discovery and validation sets
Dekker (2005)^23^	Retrospective pilot, multi-institutional (*n* = 4), The Netherlands, 1999–2005	Advanced breast, 106	54/106	Mean 52, 0:106	MRI, 65 Peptides, 87	Cytology or Neurology + MRI	Sensitivity and specificity
Fitzpatirck (2022)^24^	Prospective proof-of-concept, multi-institutional (*n* = 3) UK and Belgium, NR	Breast, 30	6/30	NR, 0:30	Cytology, 30 MRI, 29 ctDNA, 30	Diagnosis by oncology team	Sensitivity and specificity, Therapy response monitoring
Lee (2015)^25^	Prospective pilot, USA, 2005–2014	Breast, 38	21/38	Median 47.1, 0:38	CTC, 34	Cytology, or MRI, or Neurological clinical course	Sensitivity and specificity
Lin (2017)^6^	Prospective, USA, 2013–2015	Breast, 36 Lung, 31 Others, 28	30/95	Median 58, 28:67	Cytology, 95 MRI, 95 CTC, 95	Cytology, or MRI within a month	Optimal cut-off of CSF-CTC/ml, Sensitivity and specificity, positive predictive and negative predictive values
Liu (2021)^26^	Prospective pilot, China, 2012–2018	Lung adenocarcinoma with EGFR mutation.79	26/79	NR, 39:40	ctDNA, 79	MRI and cytology	EGFR mutation status, prediction of survival
Nayak (2013)^27^	Prospective pilot, USA, 2008–2010	NSCLC, 21, Breast, 15 Others, 15	15/51	NR, NR	CTC, 51	Cytology, or MRI within a month, or Physician’s diagnosis within a month	Sensitivity and specificity
Subira (2012)^28^	Retrospective pilot, Multi-institutional (*n* = 19), Spain, 2009–2010	Breast, 45 Lung, 24 Others, 5 Unknown, 4	49/78	Median 57, 24:54	Cytology, 78 CTC, 78	Cytology, or MRI, or CSF biochemical data	Sensitivity and specificity, negative and positive predictive values.
Subira (2015)^5^	Retrospective, Multi-institutional (*n* = 14), Spain, 2011–2012	Epithelial-cell solid tumors, 144	94/144	NR, NR	Cytology, 144 MRI, 129 CTC, 144	Cytology, or Clinical course + MRI + Biochemical data	Sensitivity and specificity, negative and positive predictive values, Prognostic value.
Torre (2020)^29^	Retrospective proof-of-concept pilot, Multi-institutional (*n* = 2), USA, 2016–2019	Breast, 12 Lung, 4 Others, 4	9/20	Mean 57.6, 2:18	Cytology, 20 MRI, 20 CTC, 20	Cytology, or MRI within a month	Sensitivity and specificity
van Bussel (2020)^30^	Prospective cohort, The Netherlands, 2012–2018	Breast, 28 Lung, 26 Others, 18	33/72	Mean 58, 19:53	Cytology, 72 CTC, 72 ctDNA, 10	Cytology, or MRI, or Progressive neurologic symptoms, diagnosis by a neurologist	Sensitivity and specificity, EGFR mutations in NSCLC

CTC, circulating tumor cells; ctDNA, cell-free tumor DNA; LM, leptomeningeal metastasis; MRI, magnetic resonance imaging; NR, not reported; NSCLC, non-small cell lung cancer.

### ROB and Applicability Assessment

Table 2 summarizes the assessment for ROB and applicability based on QUADAS-2. The ROB for patient selection domain was rated as low (*n* = 2) or unclear (*n* = 4) in case of unclear patient inclusion or exclusion. For the index test, half of the studies (*n* = 5) were rated as low or unclear due to unclear blinding to the results of the reference standards. The cut-off was rarely pre-specified; however, this was not downrated, since there was no widely accepted threshold for a liquid biopsy or MRI diagnosis. The reference standard was generally cytology and/or clinic-radiologic follow-up, and was rated as unclear (*n* = 5) due to unclear blinding of the results of the index tests. The interval time between the index and reference tests was not fully reported in each study, but this was not downrated since the confirmation of the date is not always possible for the final diagnosis based on the clinical course. The applicability of the index test was rated low in four studies due to no use of the leakage correction or to no blinding of the reference results.

**Table 2.** Summary of risk of bias and applicability based on QUADAS-2

Author (year)	Risk of bias				Applicability concerns		
	Patient selection	Index test	Reference standard	Flow and timing	Patient selection	Index test	Reference standard
Dekker (2005)^23^	**High**	**High**	**Low**	**High**	**High**	**High**	**Low**
Fitzpatric (2022)^24^	Unclear	**Low**	**Low**	**Low**	**Low**	**Low**	**Low**
Lee (2015)^25^	**High**	Unclear	Unclear	**High**	**High**	**High**	**Low**
Lin (2017)^6^	**Low**	**High**	**Low**	**Low**	**Low**	**High**	**Low**
Liu (2021)^26^	Unclear	**High**	**Low**	**Low**	Unclear	**High**	**Low**
Nayak (2013)^27^	Unclear	**Low**	Unclear	**Low**	Unclear	**Low**	**Low**
Subira (2012)^28^	**Low**	**Low**	Unclear	**Low**	**Low**	**Low**	**Low**
Subira (2015)^5^	Unclear	**Low**	Unclear	**Low**	Unclear	**Low**	**Low**
Torre (2020)^29^	**Low**	**Low**	**Low**	**Low**	**Low**	**Low**	**Low**
van Bussel (2020)^30^	**Low**	Unclear	Unclear	**Low**	**Low**	Unclear	**Low**

### The Meta-analysis for Detection Accuracy

Overall, the pooled prevalence of LM was 50.9% (340/668). Table 3 shows the pooled sensitivity, specificity and DOR for each index test.

**Table 3.** Synthesized sensitivity, specificity, DOR and likelihood ratios for the index tests

Index test	Number of studies	Number of patients with LM/suspected LM	Sensitivity (95% CI)	Specificity (95% CI)	DOR (95% CI)	Positive LR (95% CI)	Negative LR (95% CI)
CTC	7	251/493	0.870 (0.779–0.926)	0.938 (0.869–0.972)	100.66 (29.38–344.09)	13.973 (6.237–31.303)	0.139 (0.078–0.247)
ctDNA	2	50/109	0.979 (0.193–1.0.)	0.890 (0.253–0.995)	369.2 (0.180–7.56 × 10^5^)	8.904 (0.491–161.377)	0.024 (0.000–5.136)
MRI	5	191/338	0.694 (0.607–0.769)	0.976 (0.773–0.998)	93.3 (8.42–1034.05)	29.272 (2.655–322.695)	0.314 (0.243–0.105)
Cytology	6	239/439	0.719 (0.574–0.829)	NA	NA	NA	NA

CI, confidence interval; CTC, circulating tumor cells; ctDNA, cell-free tumor DNA; DOR, diagnostic odds ratio; LM, leptomeningeal metastasis; LR, likelihood ratio; MRI, magnetic resonance imaging; NA, not appropriate.

#### CTC

**Figure 2.**
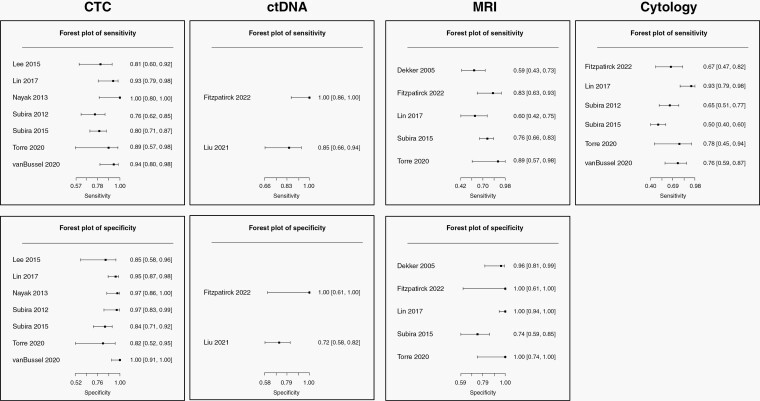
Forest plots of sensitivity and specificity across included studies of each index test and cytology. CTC, circulating tumor cell; ctDNA, cell-free tumor DNA; MRI, magnetic resonance imaging.

**Figure 3.**
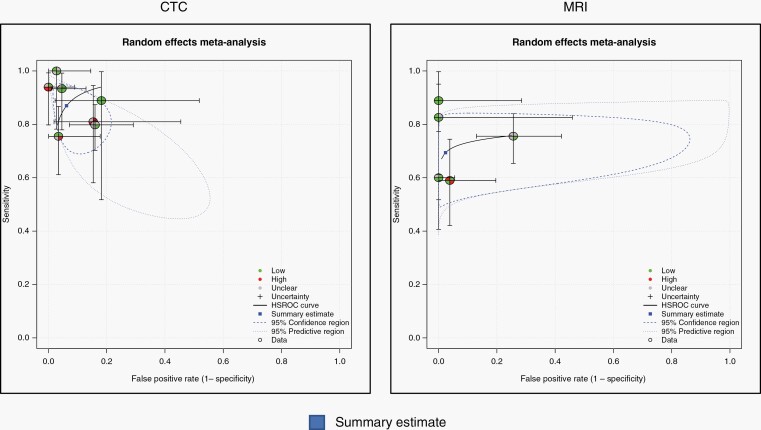
Hierarchical summary receiver operating characteristic curve displaying the estimate of sensitivity and specificity with 95% confidence intervals and the summary estimate point.

Seven studies focused on CTC as a biomarker for 493 patients with solid cancer suspected of having LM.^5,6,25,27–30^ Studies were mostly performed for patients with breast and lung cancers: 1 study was only for breast cancer, and 5 studies involved 38–60% patients with breast cancer and 20–36% patients with lung cancer. Different EpCAM-based immunoflow cytometry techniques were employed, with the CellSearch^TM^ -kit utilized in four of the studies. The pooled sensitivity and specificity were 87.0% (95% CI 77.9–92.6%) and 93.8% (86.9–97.2%), respectively. The DOR was 100.6 (29.38–344.09) (Table 3, Figure 2). The summary estimate on SROC was appropriately high as shown in Figure 3. AUC and Partial AUC were 0.931 and 0.802, respectively.

#### ctDNA

Only two studies addressed the performance of ctDNA for the diagnosis of LM in 109 patients.^24,26^ The pooled sensitivity and specificity were 97.9% (95% CI 19.3–100%) and 89.0% (25.3–99.5%), respectively. The DOR was 369.2 (0.18–7.56 × 10^5^) (Table 3, Figure 2). We did not synthesize an SROC curve because of the paucity of the included studies concerning ctDNA.

#### MRI

There were five studies including 338 patients that examined the performance of MRI.^5,6,23–29^ Imaging was performed with contrast medium before lumbar puncture, but no clear information was obtained concerning the strength of magnetic fields or sequences utilized. The pooled sensitivity and specificity were 59.4% (95% CI 60.7–76.9%) and 97.6% (77.3–99.8%), respectively. The DOR was 93.3 (8.42–1034.05) (Table 3, Figure 2). The summary estimate on SROC is shown in Figure 3. AUC and Partial AUC were 0.79 and 0.696, respectively.

#### Cytology

We detected six of 10 eligible studies presenting the performance data of cytology in 439 patients.^5,6,24,28–30^ The pooled sensitivity was 71.9% (95% CI 54.7–82.9%) (Table 3, Figure 2). The specificity and DOR could not be calculated, as positive cytology was the main reference standard for the final LM diagnosis in the articles.

## Discussion

In this systematic review and meta-analysis, four biomarkers were analyzed for accuracy at the initial diagnosis of LM in patients with solid cancers; CTC, and ctDNA, MRI, and cytology. We utilized the hierarchical model because it considers the correlation between sensitivity and specificity, accounting for within-study variability, as well as variability in effects between studies.^31^

This study shows that CTC had a pooled sensitivity of 87.0% in 493 patients with suspected LM from solid cancer. The sensitivity of CTC was better than that of CSF cytology or MRI. The specificity of CTC was sufficiently high in patients with solid cancers. The DOR of CTC was 100.6, and an SROC curve showed an appropriate point of the summary estimate (Table 3, Figure 3). There are many liquid biopsy techniques that are rapidly evolving.^13^ We need a diagnostic threshold and guidelines for the method of performing a liquid biopsy, including CTC and ctDNA for its clinical application in patients with LM from individual primary cancer.

The meta-analysis showed that the sensitivity of MRI was 69.4% in 338 patients with suspected LM from solid cancer. This sensitivity result was almost the same as that of CSF cytology (71.9%) (Table 3). The specificity of MRI was reported to be low in the literature before 2015,^5^ but has been higher in the recent reports of patients with solid cancers.^24,29^ In addition, the DOR of MRI was 93.3, and the SROC curve showed a moderate value of the summary estimate (Table 3, Figure 3).

Recent advances in clinical high-field MRI machines and sophisticated imaging sequences have resulted in the generation of high-resolution images for the routine clinical diagnosis of LM. Neuroimaging of LM presents challenges, as LM induces subtle changes in signals and enhancement in the complex shape of the space to be assessed with CSF flow or pulsation. MRI is a qualitative diagnostic tool and may have limitations of inter-reader variability and nonspecific findings^6^; however, a recent report from EORTC BTG and RANO effort showed the usefulness of a new imaging scorecard.^9,10^ Advances in technology, including the clinical usage of high-magnetic-field machines and pre- and post-contrast T2 fluid-attenuated inversion recovery (FLAIR) MRI, may refine and improve the imaging assessment of LM in the future.^32^ We should be careful when using MRI to assess various primary malignancies, as Prommel et al^33^ showed that the sensitivities of cytology and MRI were significantly influenced by the primary tumor type as solid or hematological malignancies. High-resolution imaging of the whole cranial and spinal space has the advantage of being non-invasive and is mandatory for localizing the spatial distribution of the disease.^4,6,33^ It may aid in the initial detection of LM in patients with selected solid cancers.

In the present study the sensitivity of CSF cytology was 71.9% at the first detection of LM, which was higher than previously reported.^12,24,29^ Six of the 10 eligible articles here yielded the evaluable data of CSF cytology; however, only 3 of the 6 described their precise examination protocols for cytology.^28–30^ The optimal evaluation of CSF cytology depends on an adequate CSF volume and cell counts, rapid processing, and a skilled examiner.^6,33,34^ In addition to practical issues, the sensitivity of CSF cytology had been assessed in a small-size of subjects with heterogeneous conditions thus contained outliers among the previous reports.

Our review involved an extensive search and critical review by independent authors under instruction of the PRISMA 2020 statement. The meta-analysis was done using the hierarchical model. However, several limitations should be mentioned. A major limitation is that most of the studies have a limited sample size. The reference diagnosis of LM was based on the combinations of clinical features, MRI, cytology and CSF laboratory findings, and the test modalities and methods varied among institutions, leading to a high ROB. We were unable to find studies assessing the diagnostic accuracy of the combinations of cytology and MRI, cytology and a liquid biopsy, or MRI and a liquid biopsy.

To conclude, we showed that CTC had better sensitivity than cytology, and that both CTC and MRI had appropriate specificity and DOR values for the clinical diagnosis of LM based on a meta-analysis of the diagnostic accuracy. CSF liquid biopsy and MRI may facilitate a less-invasive and earlier diagnosis, being complementary to CSF cytology. It would be beneficial to have a biomarker-driven approach to LM and a way to perform a less-invasive quick evaluation of LM, including facilitation of clinical trials for LM that is of increasing importance in the era of targeted cancer therapy. Further research is required to specify the threshold values and standards for individual primary cancers.

## Supplementary Material

vdad002_suppl_Supplementary_Material
