# Supplementary material for: Diagnostic accuracy of cerebrospinal fluid liquid biopsy and MRI for leptomeningeal metastases in solid cancers: A systematic review and meta-analysis
Source: Neurooncol Adv. 2023 Mar 5;5(1):vdad002. doi: 10.1093/noajnl/vdad002 (PMC10034915; doi:10.1093/noajnl/vdad002)
Supplement: vdad002_suppl_Supplementary_Material [file vdad002_suppl_supplementary_material.docx]

**Supplemental data 1**

*Search for PubMed*

#1 Meningeal Carcinomatosis[mh] OR Meningeal Neoplasms[mh] OR "Central Nervous System Neoplasms/cerebrospinal fluid"[mh]=26,908

#2 "Meningeal Carcinomatosis"[tiab] OR "Meningeal Carcinomatoses"[tiab] OR "Carcinomatous Meningitis"[tiab] OR "Leptomeningeal Carcinomatosis"[tiab] OR "Leptomeningeal Carcinomatoses"[tiab] OR "leptomeningeal metastasis"[tiab] OR "leptomeningeal metastases"[tiab] OR "leptomeningeal disease"[tiab] OR "neoplastic meningitis"[tiab]=2,965

#3 #1 OR #2=28,088

#4 metastasis[tw] OR metastases[tiab] OR metastase[tiab] OR Meningeal Carcinomatosis/ secondary[mh] OR Meningeal Neoplasms/secondary[mh] OR Central Nervous System Neoplasms/ secondary[mh] OR "cerebrospinal fluid"[sh] OR "cerebrospinal fluid"[tiab] OR CSF[tiab]=665,298

#5 #3 AND #4=7,908

#6 Hematologic Neoplasms[mh] OR Lymphoma[mh] OR Leukemia[mh] OR Multiple Myeloma[mh] OR meningioma[mh]=476,844

#7 #5 NOT #6=4,457

#8 diagnosis[sh] OR diagnostic imaging[sh] OR diagnosis[tiab] OR diagnose[tiab] OR diagnosed[tiab] OR diagnosing[tiab] OR diagnostic[tiab] =5,298,764

#9 "Neoplastic Cells, Circulating"[mh] OR "Circulating Tumor Cells"[tiab] OR "Circulating Tumor Cell"[tiab] OR "CTC"[tiab] OR "CTCs"[tiab] OR "Neoplasm Circulating Cells"[tiab] OR "circulating neoplastic cell"[tiab] OR "circulating neoplastic cells"[tiab] OR "tumor embolism"[tiab] OR "tumor embolisms"[tiab] =19,593

#10 "Cell-Free Tumor DNA"[tiab] OR cfDNA[tiab] OR ctDNA[tiab] OR "Circulating Tumor DNA"[tw] =7,730

#11 Liquid Biopsy[tw] OR "Biopsies Liquid"[tiab] OR "Biopsy Liquid"[tiab] OR "Liquid Biopsies"[tiab] =6,361

#12 "Magnetic Resonance Imaging"[mh] OR "Magnetic Resonance"[tiab] OR MRI[tiab] =731,936

#13 cytology[tiab] OR cytologic[tiab] OR pathology[tiab] OR pathologic[tiab] OR pathologist[tiab] OR Meningeal Carcinomatosis/cytology[mh] OR Meningeal Neoplasms/cytology[mh] OR Meningeal Carcinomatosis/pathology[mh] OR Meningeal Neoplasms/pathology[mh] =581,134

#14 #8 OR #9 OR #10 OR #11 OR #12 OR #13=5,945,867

#15 #7 AND #14=3,079

#16 #15 AND ("2000/01/01"[dp]:"2022/01/09"[dp]) AND english[la] =**1,855**

**Supplemental data 2**

*Search for Cochrane Central*

#1 ("Meningeal Carcinomatosis" OR "Meningeal Carcinomatoses" OR "Carcinomatous Meningitis" OR "Leptomeningeal Carcinomatosis" OR "Leptomeningeal Carcinomatoses" OR "leptomeningeal metastasis" OR "leptomeningeal metastases" OR "leptomeningeal disease" OR "neoplastic meningitis"):ti,ab,kw =130

#2 ((Meningeal OR Leptomeningeal) NEAR (Carcinomatos?s OR metastas?s OR disease OR diseases)):ti,ab,kw =132

#3 ((Carcinomatous OR neoplastic) NEAR/3 Meningitis):ti,ab,kw =46

#4 [mh "Meningeal Carcinomatosis"] OR [mh "Meningeal Neoplasms"] OR [mh "Central Nervous System Neoplasms"/CF] =131

#5 #1 OR #2 OR #3 OR #4 =270

#6 [mh metastasis] OR metastas*:ti,ab,kw =27141

#7 [mh "Meningeal Carcinomatosis"/SC] OR [mh "Meningeal Neoplasms"/SC] OR [mh "Central Nervous System Neoplasms"/SC] =373

#8 [mh /CF] OR ("cerebrospinal fluid" OR CSF):ti,ab,kw =10357

#9 #6 OR #7 OR #8 =37255

#10 #5 AND #9 =149

**Supplemental data 3**

*Search for SCOPUS*

#1 TITLE-ABS-KEY ( ( ( carcinomatous  OR  neoplastic )  W/3  meningitis )  OR  "leptomeningeal disease" )  OR  ( ( meningeal  OR  leptomeningeal )  W/3  ( carcinomatos?s  OR  metastasis  OR  neoplasms ) ) ) =17,807

#2 TITLE-ABS-KEY ( ( metastas*  OR  "cerebrospinal fluid"  OR  csf ) ) =969,772

#3 #1 AND #2=6,722

#4 TITLE-ABS-KEY ( "Hematologic Neoplasms"  OR  lymphoma  OR  leukemia  OR  "Multiple Myeloma"  OR  "meningioma" ) =789,007

#5 #3 NOT #4=3,330

#6 TITLE-ABS-KEY ( diagnos* ) =5,544,718

#7 TITLE-ABS-KEY ( ( "Neoplastic Cells"  OR  "Neoplastic Cell"  OR  "Tumor Cells"  OR  "Tumor Cell"  OR  "Tumour Cells"  OR  "Tumour Cell" )  W/3  circulating ) =14,611

#8 TITLE-ABS-KEY ( ctc  OR  ctcs ) =16,131

#9 TITLE-ABS-KEY ( ( ( neoplasm  OR  neoplastic  OR  tumor  OR  tumour )  W/3  ( "Circulating Cells"  OR  "Circulating Cell" ) ) ) =4,059

#10 TITLE-ABS-KEY ( ( ( tumor  OR  tumour )  W/3  ( embolism  OR  embolisms ) ) ) =8,338

#11 TITLE-ABS-KEY ( ( "cell Free"  OR  circulating )  W/3  ( "Tumor DNA"  OR  "Tumour DNA" ) ) =5,300

#12 TITLE-ABS-KEY ( ( "cell-Free" )  W/3  ( "Tumor DNA"  OR  "Tumour DNA" ) ) =473

#13 TITLE-ABS-KEY ( cfdna  OR  ctdna ) =5,964

#14 TITLE-ABS-KEY ( ( liquid  W/3  ( biopsy  OR  biopsies ) ) ) =8,154

#15 TITLE-ABS-KEY ( "Magnetic Resonance Imaging"  OR  "Magnetic Resonance"  OR  mri ) =1,615,543

#16 TITLE-ABS-KEY ((cytology  OR  cytologic  OR  pathology  OR  pathologic  OR  pathologist ) ) =3,018,829

#17 #6 OR #7 OR #8 OR #9 OR #10 OR #11 OR #12 OR #13 OR #14 OR #15=74,649,784

#18 #5 AND #17=2,668

#19 PUBYEAR  >  1999 =8,834,457

#20 #18 AND #19 =2,098

#21 LANGUAGE ( english  OR  japanese ) =55,106,434

#22 #20 AND #21=1,974

#23 DOCTYPE ( ar  OR  re  OR  ch ) =67,180,189

#24 #22 AND #23=1,786
